# Immune System‐Related Genetic Risk Factors for Inhibitory Antibody Development in Patients With Hemophilia: Reviewing an Old Problem From a New Perspective—A Narrative Review

**DOI:** 10.1002/hsr2.71331

**Published:** 2025-10-06

**Authors:** Fatemeh Zeylabi, Mojtaba Aghaei, Najmaldin Saki

**Affiliations:** ^1^ Cellular and Molecular Research Center, Basic Health Sciences Institute Shahrekord University of Medical Sciences Shahrekord Iran; ^2^ Student Research Committee Ahvaz Jundishapur University of Medical Sciences Ahvaz Iran; ^3^ Thalassemia & Hemoglobinopathy Research Center, Health Research Institute Ahvaz Jundishapur University of Medical Sciences Ahvaz Iran

**Keywords:** hemophilia, immune system, inhibitor, inhibitory antibody

## Abstract

**Background and Aims:**

Hemophilia A and B are two of the most common bleeding disorders. Genetic risk factors are associated with the development of autoantibodies released in hemophilia patients against alternative factors and are the most important problems associated with the care of these patients.

**Objective:**

In this study, we reviewed genetic risk factors related to the immune system in patients with hemophilia A and B who developed inhibitory antibodies against factors 8 or 9.

**Methods:**

This study is based on the PubMed database and Google Scholar search engine information (2016–2025) in English using the terms “inhibitors”, “inhibitory antibodies”, “immune system”, and “hemophilia”.

**Results:**

Studies have shown that multiple genetic factors (CTLA‐4, PTPN22, and cytokine polymorphisms) increase the risk of producing an inhibitor against alternative factors (8 and 9) in patients with severe hemophilia.

**Conclusions:**

The presence of inhibitory antibodies in patients with severe hemophilia may be associated with immune system‐related genetic risk factors, and several studies have shown that in most cases, immune system‐related polymorphisms (rs2476601 PTPN22, rs2069812 IL5, and rs1800629 TNF‐α) produce autoimmune antibodies or exacerbate them.

## Summary

- Approximately 25% of patients with severe hemophilia A develop antibodies against factor 8, and approximately 1.5%–3% of all patients with hemophilia B develop antibodies against factor 9.
- Genetic risk factors play essential roles in the development of inhibitory antibodies.
- Autoimmune‐related genetic factors (Bruton tyrosine kinase, FC gamma receptors, and PTPN22) and cytokines (IL2, IL4, IL5, and IL10) are involved in the development of inhibitory antibodies.

## 1 Introduction

Hemophilia A is the most common bleeding disorder, affecting 1 in 5000 people in the general population, and hemophilia B affects 1 in 3000 individuals [1]. Hemophilia A and B are inherited and congenital bleeding disorders caused by mutations in coagulation factors 8 and 9 [2]. There are mild to severe forms of the disease, but in most cases, patients experience recurrent bleeding in large joints, such as the elbows, knees, and ankles [2, 3]. The number of hemophilia patients worldwide is estimated to be approximately 40,000. This number includes all cases of mild to severe hemophilia A and B. Currently, developing alloantibodies against injectable factors 8 and 9 is one of the most critical problems in treating patients with severe hemophilia [4, 5, 6]. People who produce antibodies against recombinant factors 8 and 9 are classified as severe hemophilia patients. Approximately 25% of people with severe hemophilia A secrete antibodies against factor VIII (FVIII), and approximately 1.5%–3% of all patients with hemophilia B secrete antibodies against factor IX (FIX) [2, 7]. Self‐reactive T cells can cause autoimmune diseases directly or via interactions with their targets. Additionally, B lymphocytes contribute to the progression of the disease by producing antibodies. The emergence of antibodies in inhibitory patients is due to numerous events, such as immune mechanisms [1, 2]. Numerous factors control the severity and extent of symptoms in this disease, such as the type of injectable factor, injectable dose, and genetic risk factors (such as HLA molecules and CTLA4), which are the most critical factors. The leading cause of hemophilia is a mutation in the F8 gene gene (in hemophilia A) and F9 gene (in hemophilia B) [3, 8, 9]. More than 900 mutations have been identified in the coding region of the factor VIII (FVIII) gene, which supports genetic heterogeneity in hemophilia [10].

Modulators of innate immunity are upregulated and may act as persistent danger signals and influence the responses and ultimate outcomes of immune tolerance induction therapy. Results of studies show that in patients with active inhibitors, the expression of genes associated with the innate immune system, including NLRP3 and TLR8, which promote inflammation, is increased. These findings indicate that the innate immune response plays a key role in the formation of inhibitors and the effectiveness of immune tolerance therapies [11].

On the other hand, the risk of inhibitor antibodies (inhibitors) is greater in patients with a family history of the disease, so this condition is more common in siblings and family members who have the same mutations [12, 13]. Despite advances in the treatment of these patients, some therapeutic procedures, such as increasing immune tolerance, are not always effective and, in many cases, impose a high cost on patients; thus, finding diagnostic biomarkers is essential for the early diagnosis of these diseases [4]. Many studies have been published on various aspects of hemophilia worldwide, but the mechanisms and factors causing this condition remain unclear. Hemophilia A patients who develop inhibitors to FVIII therapy face significant treatment challenges. Studies emphasize that, in addition to genetic and environmental factors, complex regulatory pathways are involved in the development of inhibitors. Attention to these mechanisms could pave the way for new therapeutic targets, especially in a situation where current strategies such as immune tolerance induction are often lengthy, costly, and ineffective [14].

Although the antibodies produced in hemophilia inhibitor patients are alloantibodies, in this study, we aimed to collect and classify more consistent genetic factors related to the immune system in these patients for better understanding of the disease and to improve disease management.

## 2 Classification and Clinical Findings of Hemophilia Patients

Hemophilia is classified according to the plasma factor level and the severity of clinical symptoms. Patients with plasma factor levels less than 1 IU/dL are classified as having severe hemophilia. Individuals with 1–5 IU/dL and those with more than 5 IU/dL are classified as moderate and mild, respectively [15, 16]. Approximately 35% of patients with Hemophilia A and 29% of those with Hemophilia B are classified as having severe disease [17].

Approximately 30%–40% of hemophilia B patients and less than 1% of hemophilia A patients are considered severe forms [1, 2]. Patients with mild to moderate forms of hemophilia are diagnosed mainly in the later years of life, in childhood and adolescence, and most often with an average age of 11.5 years. Approximately 25% of patients with severe hemophilia A develop antibodies against VIII factor, and approximately 1.5%–3% of all hemophilia B patients develop antibodies against IX factor [2, 7, 18]. Patients with hemophilia A and B usually tend to bleed on the basis of the level of deficiency in VIII (FVIII) and IX factors. Although patients with severe forms show a severe bleeding phenotype, a percentage of these individuals experience mild bleeding. There are also people with more than 5 IU/DL VIII (FVIII) and IX factors who tend to bleed profusely [15, 16]. Patients with mild hemophilia only bleed profusely when they experience significant injuries or undergo major surgery. However, spontaneous bleeding or bleeding following minor injuries occurs in the severe form of the disease. Severe hemophilia is characterized by hemarthrosis, soft tissue hematoma, and recurrent bleeding.

Hematomas are associated with arthropathy, muscle contractions, and pseudotumors with chronic pain [16, 19]. Patients who produce antibodies against alternative factor inhibitors are classified as having severe hemophilia [2]. The inhibitors are high‐affinity polyclonal immunoglobulins secreted against alternative coagulation factors, and this process is dependent on T cells, antigen‐supplying cells, helper T lymphocytes, and B lymphocytes [15]. Inhibitors are classified on the basis of the alternative factor's kinetics and degree of inhibition. Type 1 inhibitors are linear, dose‐dependent inhibitors that inhibit the replacement factor entirely. These types of inhibitors are common in severe hemophilia. Type 2 inhibitors have complicated kinetics, inactivate the replacement factor, and are more common in mild forms of the disease [20]. Inhibitory antibodies have been developed against the factor A2, A3, and C2 domains. The causes of the development of these antibodies are still unknown, but studies have shown that various genetic and environmental risk factors are among the important risk factors for this disease [6, 21].

## 3 Genetic Risk Factors in Hemophilia Patients With Inhibitory Antibodies

### 3.1 Cytotoxic T Lymphocyte‐Associated Protein 4

**Figure 1:**
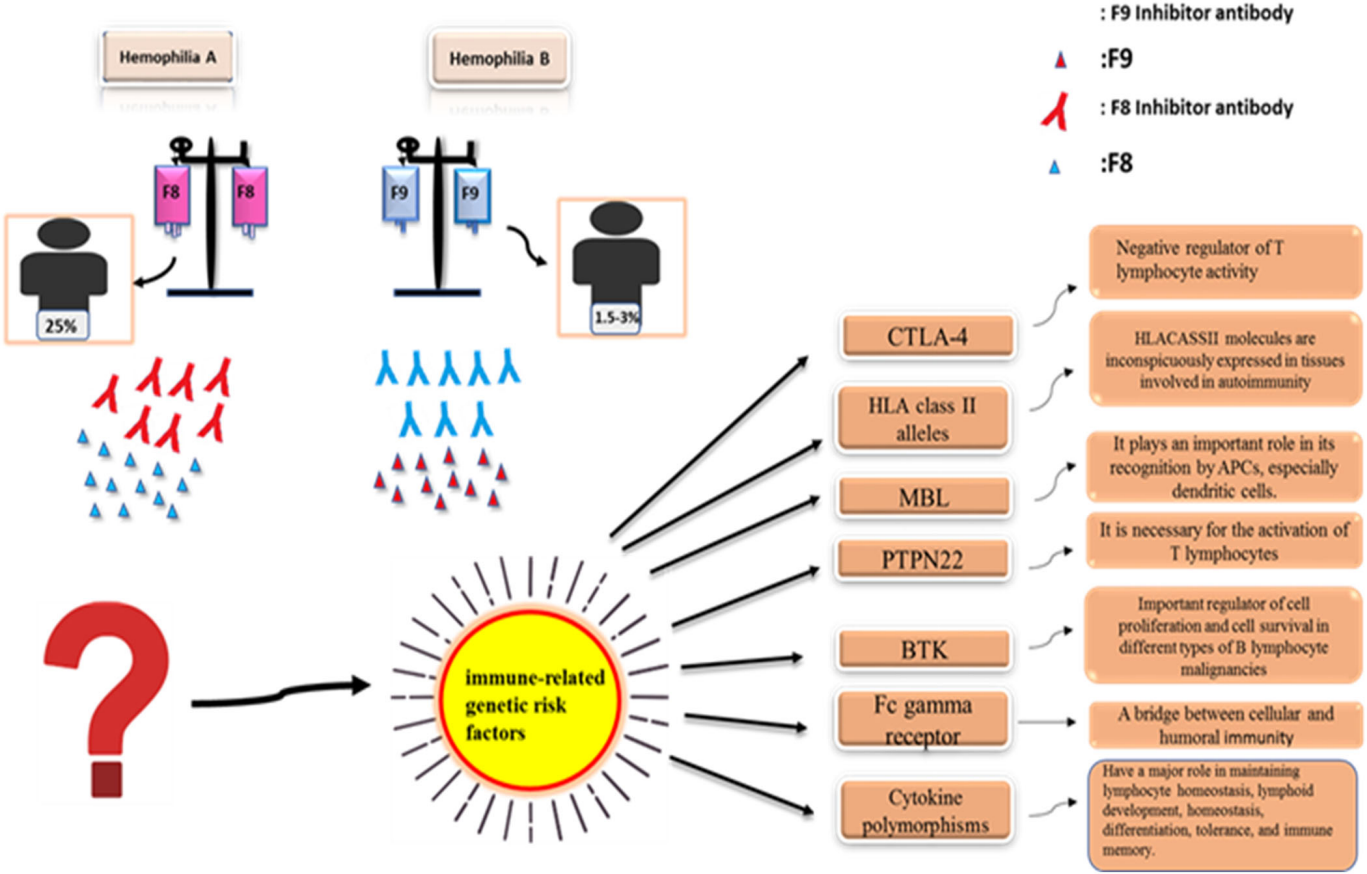
Hemophilia patients A and B are deficient in coagulation factors 8 and 9, respectively. Hemophilia inhibitors produce antibodies against injectable alternative factors. multiple genetic factors (CTLA‐4, PTPN22, Cytokine polymorphisms, …) increase the risk of producing an inhibitor Ab against an alternative factors.

Producing inhibitors against alternative factors requires the cooperation of immune system cells, including helper T cells, antigen‐presenting cells (APCs), and active B and T lymphocytes. CTLA‐4 is a T lymphocyte cytotoxic antigen that is presented on the surface of active T cells and negatively regulates the activity of these lymphocytes by competing with CD28 for binding to B7, regulating T‐cell activity, and affecting cell adhesion [22]. Blocking the activity of CTLA‐4 can increase the activity of T cells and, thus, the production of antibodies. On the other hand, regulatory T cells (Tregs) play an essential role in the development of tolerance in inhibitory patients during factor injection. CTLA‐4 has recently been identified as an essential membrane protein necessary for the suppressive function of Tregs, and CTLA‐4 reduces the phosphorylation of MAPK under T‐cell stimulation conditions and limits the function of Smad2/3, which is a crucial transcription factor for FOXP3. Therefore, deletion of CTLA‐4 or mutations in this gene and related polymorphisms can lead to loss of immune tolerance and autoimmune disease in humans. Several mutations in the CTLA‐4 gene have been identified, leading to recurrent infectious diseases, hypogammaglobinemia, autoimmune diseases, and lymphocytic infiltration. Further studies have shown that individuals with CTLA‐4 deficiency have a variety of homeostasis system‐related diseases [22, 23]. Studies have indicated that polymorphisms in the promoter region of CTLA‐4 may be associated with an increased risk of antibody production against alternative therapeutic factors in patients with hemophilia A and B. A significant association exists between single nucleotide polymorphisms (SNPs) at position 318 of the CTLA‐4 gene promoter and antibody production in patients with hemophilia A inhibition. T nucleotides at this position increase the susceptibility of patients to developing inhibitors. This polymorphism is also associated with Hashimoto's disease, multiple sclerosis (MS), Graves’ disease, and Wegener's granulomatosis (Figure 1) [24, 25].

### 3.2 HLA Class II Alleles

T cells play an essential role in killing pathogens in infected cells and trigger the production of different types of antibodies to protect against exogenous microorganisms and toxic molecules. To perform these critical functions, T cells must communicate directly with other cells, including APCs and major histocompatibility complex (MHC, also known as human leukocyte antigen (HLA) molecules, and then find pathogenic antigen threats. HLA‐related polymorphisms are another essential factor that can increase the risk of inhibitory antibody production in hemophilia patients. The wide diversity among individuals can be closely linked to autoimmune diseases [7, 26, 27, 28]. It is well known that HLA class II molecules are imperceptibly expressed in tissues involved in autoimmunity. Additionally, proteins that are not processed correctly in the endoplasmic reticulum and have escaped proteasome degradation are transported to the cell surface via MHC class II without peptide processing. Because these proteins are different from natural proteins, they also have different antigenicities. These proteins and the MHC that present them are the targets of antibody development in autoimmune diseases such as systemic lupus erythematosus (SLE), rheumatoid arthritis (RA), and antiphospholipid syndrome; thus, these proteins are likely self‐proteins, and antibodies are developed against them. On the other hand, a strong correlation has been observed between protein binding to HLA, which indicates that these molecules and their related alleles, especially HLA‐DR, are effective in pathogenicity or severity. Unlike other HLA‐DRB1 alleles, which were not significantly different from those in the control group, the DRB1 * 1501 polymorphism of the HLA Class II gene was observed in more inhibitory patients and was significantly different from that in the control group. The DQB1*06:02 polymorphism was significantly observed in individuals with hemophilia A inhibition at the DQ gene locus. The DQB1*06:02 and DRB1*15 alleles are strongly associated with patients with severe hemophilia in Caucasian populations, and this allelic distribution may vary across different populations and ethnicities. These ethnic differences are so crucial in HLA alleles that the presence of these alleles exacerbates bleeding in ethnicities. Some studies have shown that these alleles are significantly related to patients with an intron 22 inversion; otherwise, no significant relationship has been observed [7, 26].

### 3.3 Mannose‐Binding Lectin (MBL)

MBL is a soluble pattern recognition receptor that can detect antigens and participates in nonspecific cellular immunity, such as regulating inflammation, cell migration, opsonization, phagocytosis, and cell deletion, which plays an essential role in innate immunity. MBL has been demonstrated to be a genetic risk factor for autoimmune diseases such as MS, Guillain–Barré syndrome, and myasthenia gravis (MG) [29, 30, 31]. Although complement has been shown to play an essential role in the pathogenesis of MS, GBS, and MG, the role of MBL as an indicator of the immunopathogenesis of these diseases and its relationship with their severity has been emphasized. Studies have shown that the mannosylation of F8 plays an essential role in its detection by APCs, especially dendritic cells (DCs). The mannose receptor at the macrophage and DC cell surface is CD206. MBL2 polymorphisms with B alleles were observed only in inhibitory patients [9, 32, 33].

### 3.4 Protein Tyrosine Phosphatase (PTP), Nonreceptor Type 22

The lymphoid‐specific tyrosine phosphatase protein 22 (PTPN22) is encoded on chromosome 1 (p13.3–p13.1) and has a phosphatase N‐terminal domain and a C‐terminal domain with proline motifs. T lymphocyte activation requires the activity of phosphatases and intracellular proteases after they interact with receptors and epitopes. One of the essential phosphatases involved in regulating T lymphocyte activity is PTP, which is encoded by the PTPN22 gene. The nanoreceptor two tyrosine phosphatase protein‐encoding gene on chromosome 1 has been identified as an autoimmune gene in many autoimmune diseases [34, 35]. The PTPN22 Rs2476601 polymorphism (exon 14 c.1858C> T, p.Arg620Trp) is a functional variant that is strongly associated with various autoimmune diseases (such as vitiligo and SLE, Type 1 diabetes, RA, and Graves), disorders of T‐cell regulation and antibody formation. This variant is a single nucleotide change (cytidine to thymidine) that converts the amino acid arginine to tryptophan at position 620 of the PTPN22/Lyp protein (Figure 1) [36, 37, 38, 39, 40, 41, 42].

### 3.5 Bruton Tyrosine Kinase (BTK)

BTK is a significant B‐cell receptor (BCR) signaling component. It is an essential regulator of cell proliferation and survival in various B lymphocyte malignancies. B lymphocytes play an important role in systemic autoimmune diseases in which the production of autoantibodies is significant, such as RA, SLE, and Sjogren's syndrome (SS). In primary SS, circulating nuclear antibodies and germ cells (GCs) in the salivary glands are associated with disease severity. Further studies indicated that genes involved in B‐cell activation and differentiation are involved in the pathogenesis of autoimmune diseases. Additionally, B cells are precursors of plasmablast and plasma cells (PCs). They can activate T cells by supporting the differentiation of follicular T lymphocytes, the antigen supply, and the production of inflammatory cytokines [43, 44]. BTK activation is mediated by cell membrane binding and Y551 phosphorylation of the second kinase and the SRC kinase family, or splenic tyrosine kinases (SYKs), which initiates its catalytic activity. As a result of these events, Y223 autophosphorylation occurs in the SH3 domain [45]. BTK is found in innate immune system cells and activates phosphorylate and phospholipase Cγ (PLCγ), which leads to an increase in Ca^2+^ and activation of the NF‐κB and MAP kinase pathways. Mutation of BTK in humans causes inherited X‐linked agammaglobulinemia, which is characterized by B‐cell deficiency and low levels of Ig in the serum. This enzyme stimulates the response of the BCR to the antigen and stimulation via CD40, TLRs, FC receptors, and chemokine receptors, so adult B cells are dependent on this enzyme for its activities. Evidence suggests that BTK is also present in the myeloid lineage and participates in other signaling pathways but is known mainly as the selective restriction kinase of B lymphocytes. The use of an anti‐CD20 antibody (rituximab) to eliminate mature B lymphocytes in clinical trials has well demonstrated the role of these cells in autoimmune diseases such as RA, lupus, and MS. Importantly, BTK modulates signaling such that its overexpression causes autoimmunity. Decreased expression reduces autoimmunity, and proper regulation of the BTK protein is essential for maintaining immune tolerance [43, 46, 47]. Precise control of B‐cell differentiation into PCs is crucial for eliciting appropriate immune responses and preventing autoimmunity. The Ets1 transcription factor in B cells prevents PC differentiation and is expressed by activated B cells. It is regulated by BCRs and TLRs and is maintained by Lyn‐, CD22‐, Siglec G‐, and SHP‐1‐mediated inhibitory signals. In the absence of these inhibitory elements, the level of Ets1 in B cells decreases in a BTK‐dependent manner. All of these events eventually lead to the production of PCs and, eventually, the production of autoantibodies in autoimmune diseases [45]. These studies show that the use of BTK inhibitors can be helpful in the treatment of autoimmune diseases. Studies suggest that inhibition of BTK via PF‐06250112 inhibits memory B cells in response to factor eight substitutes in hemophilia A patients [47, 48].

### 3.6 The FC Gamma Receptor (FcγR)

Antibodies bind to microorganisms through their receptors and to FC receptors on the surface of leukocytes through their FC (at the carboxyl end). FC receptors are glycoproteins widely expressed at the surface of the hematopoietic system (monocytes, macrophages, and IFN‐γ‐stimulated neutrophils) and act as bridges between cellular and humoral immunity. These receptors bind to the FC portion of IgG. FcγRIIA and FcγRIIC are found mainly in phagocytic cells (neutrophils, monocytes, and macrophages). In contrast, FcγRIIB is expressed mainly in B lymphocytes and especially in phagocytic leukocytes to negatively regulate cell functions, such as phagocytosis. Human FcγRIIA is a specific receptor that does not have FcRγ‐related chains. FcγRIIA contains ITAM in its cytoplasmic region, whereas FcγRIIB has a different tyrosine‐containing motif in negative signaling. FcγRIIIA is expressed in basophils and mast cells. Crosslinking of these receptors on the leukocyte surface activates several practical cell functions. These actions destroy microbial pathogens and induce a critical inflammatory state during infection. Depending on the cell type and type of Fcγ receptor, these include phagocytosis, cell degranulation, the production of various cytokines and chemokines, cell‐mediated antibody‐dependent cytotoxicity (ADCC), and gene activation. However, in autoimmune diseases, antibodies can direct these practical functions toward damage to normal tissues and cause severe tissue problems. The IgG subclass and glycosylation pattern of antibodies are influential factors in IgG‐FcγR activity. Additionally, other molecules, such as members of the pentraxin family, can bind to these receptors [40]. At the initiation of the adaptive immune response, antibodies are of the IgM class. These antibodies are less prone to microbes but can easily activate the classical complement pathway, which in microorganisms can induce phagocytosis through complement receptors or induce bacterial lysis by forming a membrane attack complex (MAC). A secondary adaptive immune response subsequently occurs with the production of IgG, which tends to have increased affinity and specificity. Thus, dysfunction of IgG causes immunodeficiency. The FC1 131R> H polymorphism in the FCGR2A gene has indicated that individuals with the HH genotype are associated with an increased risk of inhibitory production in individuals with hemophilia A. The role of this FCYR polymorphism in autoimmune diseases has been well demonstrated [8].

### 3.7 C‐X‐C Motif Chemokine Ligand (CXCL)

Studies have shown that patients with hemophilia A who have never received factor VIII have significantly increased levels of CXCL8 and decreased levels of CXCL10 and MCP1. These changes in cytokine profiles indicate an underlying inflammatory pattern that could be associated with an increased risk of developing inhibitory antibodies after initiation of therapy [49].

An examination of the immune response of hemophilia A patients after the first factor VIII injection showed that in these patients with the inhibitor, CXCL8 and Interleukin 6 (IL6) levels were significantly increased, indicating an initial severe inflammatory response [50].

CXCL13, also a chemokine associated with T follicular helper (TFH) activity and CXCR5 ligand, has been identified as a potential biomarker for autoimmune diseases such as RA, (SLE), SS, and thrombocytopenia. CXCL13 may contribute to the interaction of endothelial cells (ECs), which are the main sites of physiological FVIII synthesis, with circulating TFHs. Studies have shown that in hemophilia A patients with inhibitors, CXCL13 levels were significantly twofold higher than in patients without inhibitors [51].

Studies have also shown that combination therapy with anti‐CD20 and CXCL13 monoclonal antibodies results in a significant reduction in the development of FVIII inhibitors, a decrease in TFH cells, and an increase in Tregs in the spleen. A sustained reduction in antibody‐producing PCs has also been observed in the bone marrow and spleen. These findings suggest that simultaneous targeting of CD20 and CXCL13 could be an effective strategy for inducing immune tolerance in inhibitor‐producing hemophilia A patients [52]. On the other hand, the results of another study showed that the balance between TFH and T follicular regulatory cells plays a decisive role in the formation of the immune response against factor VIII in hemophilia A mouse models, and an increase in the TFH/TFR ratio is directly correlated with the production of FVIII inhibitory antibodies and can be considered as a key indicator in predicting the immune response of patients. Also, deletion of TFR cells or manipulation of their regulatory pathways has led to dramatic changes in the levels of inhibitory antibodies [53].

### 3.8 Cytokine Polymorphisms

Cytokines play a significant role in maintaining lymphocyte homeostasis, lymphoid development, homeostasis, differentiation, tolerance, and immune memory under stable and even inflammatory conditions. Unregulated lymphocytes can lead to autoimmunity under stable conditions and cause extensive tissue damage during inflammation. Regulatory cytokines work with other environmental signals to regulate lymphocyte function and activation properly. Many recent studies have highlighted the importance of regulatory cytokines in controlling the differentiation and function of lymphocytes under stable and inflammatory conditions and minimizing tissue damage. IL10 is a central immune system regulator that prevents inflammatory tissue damage because it limits the inflammatory response. Additionally, IL10 is essential for homeostasis and immune system balance. CD25^+^ CD4^+^ Tregs have been shown to suppress active autoregulatory T cells via a mechanism involving IL10. CD25^+^ CD4^+^ Tregs inhibit the activation of autoreactive T cells in vitro and in vivo and suppress specific autoimmune diseases. On the other hand, in addition to the role of this cytokine in autoimmune diseases (such as Hashimoto's thyroiditis and juvenile RA), the importance of IL10 polymorphisms in increasing sensitivity to certain drugs (such as beta‐lactams) should also be considered [54, 55]. The role of TNF‐α in autoimmune diseases such as Crohn's disease and RA has been proven, and its increased level is recognized as one of the critical elements in the development of autoimmune diseases [56, 57]. TNF antagonists are among the most effective treatments for some autoimmune diseases, such as RA. Studies have shown that TNF‐α is not merely a pro‐inflammatory cytokine but also an immune regulatory molecule that can alter the balance of Tregs [56]. BAFF (B‐cell activating factor) is a cytokine of the TNF family that plays a key role in the survival and maturation of B cells. Overproduction of BAFF is associated with systemic autoimmune diseases such as lupus, as it promotes the survival of autoreactive B cells [58].

Studies have shown that BAFF levels are increased in hemophilia A patients (children and adults) who have factor VIII inhibitor antibodies. After successful immune tolerance induction (ITI), BAFF levels are reduced to a similar extent as in individuals without inhibitors. Also, in mouse models of hemophilia A, prophylactic administration of anti‐BAFF antibody before immunization with FVIII prevented the development of inhibitors; furthermore, the combination of anti‐CD20 and anti‐BAFF antibodies targeting FVIII‐specific PCs significantly reduced the presence of inhibitors. These results indicate that BAFF is a key regulator in the formation and persistence of FVIII inhibitory antibodies and that anti‐CD20/BAFF combination therapy could improve the efficacy of immune tolerance induction strategies [59].

Interleukin 2 (IL2) is a multifunctional cytokine that regulates immunity and is an essential factor in the proliferation and differentiation of T lymphocytes in the thymus. It also stimulates effective T cells to stimulate immune responses. A characteristic feature of Treg cells is the expression of high‐affinity IL2 receptors (IL2Rs), which has led to numerous approaches for treating autoimmune diseases. This cytokine is an essential factor in the functions of CD4^+^ T cells, and the functions of these cells play essential roles in the development and spread of autoimmune diseases [60, 61]. This cytokine has different biological activities, such as the ability to differentiate B and T lymphocytes, stimulate the production of acute‐phase proteins in the liver, and activate macrophages and NK cells. IL2 is produced by monocytes, macrophages, vascular ECs, and smooth muscle cells [62, 63, 64, 65]. This cytokine plays a key role in relaxing B and T lymphocytes, thus tolerating immunity [66, 67, 68]. IL 12 was initially identified and isolated as an NK cell‐stimulating factor. However, its ability to activate cytotoxic T cells, differentiate into CD41^+^ T cells, balance Types 1 and 2 lymphocyte responses, prepare macrophages for no production, produce IFN‐γ, and induce CXC‐dependent antiangiogenic activity has recently been considered [60, 68, 69, 70, 71]. Th1 cells secrete IFN‐γ, triggering delayed hypersensitivity responses and facilitating macrophage activation. These cytokines are important in many destructive autoimmune diseases, such as allergies, encephalomyelitis, Hashimoto's thyroiditis, and Type 1 diabetes [28, 72, 73, 74]. Table 1 lists the autoimmune‐related polymorphisms in the cytokines listed in inhibitor patients.

**Table 1:** Autoimmune‐related polymorphisms studied in patients with hemophilia treated with inhibitors.

Gene	Polymorphism	Role in autoimmunity	In inhibitor hemophilia	Ref.
TNF‐α	rs1800629	Type 2 diabetes	Effective in AGA haplotype	[7, 26]
IFN‐γ	+874A/T	Hashimoto thyroiditis	Ineffective or decreased	[72, 75, 76]
IL1β	rs1143627	Graves’ disease	Ineffective	[77, 78]
IL6	rs1800795(‐174G/C)	Rheumatoid arthritis Type 2 diabetes	Ineffective	[79, 80, 81]
IL2	rs2069762	Addison disease	Effective	[60, 77, 82, 83, 84, 85]
	rs4833448	Sclerosing cholangitis		
IL5	rs2069812	Graves’ disease and allergy	Effective	[86, 87]
IL4	rs2227282	Rheumatoid arthritis	Effective	[20, 23, 88, 89]
	(C‐590T)	Type 2 diabetes/autoimmune hepatitis Type 1	Ineffective	
IL10	rs6667202	Juvenile rheumatoid arthritis	Effective	[20, 27, 77]
	rs4072226	Hashimoto thyroiditis		

Abbreviations: A, adenine; C, cytosine; G, guanine; IL, interleukin; T, thymine; TNF, tumor necrosis factor.

## 4 Discussion

An improper or altered immune system response to antigens can cause autoimmune diseases. Studies have shown that genetic risk factors are critical in causing various diseases of varying severity. We know that autoimmune diseases can be associated with blood diseases or that even antibodies can be developed against components of the hematopoietic system, as previous studies have reported on immune thrombocytopenia (ITP) or autoimmune hemolytic anemia. ILs play essential roles in regulating the response of immune cells, especially B and T lymphocytes, as well as the mechanisms involved in the production of antibodies in autoimmune diseases [90]. Further studies of factors associated with the overproduction or inhibition of ILs can help assess patient prognosis or identify an appropriate treatment approach. The role of ILs such as IL1, IL2, IL4, IL5, IL6, IL10, and IL12 and their associated polymorphisms in patients with hemophilia inhibitors has been well demonstrated [18]. Polymorphisms of BTK (which play important roles in the regulation of cell proliferation, cell survival, and the development of immune tolerance), FcγRs (the bridge between cellular and humoral immunity), PTPN22 (which is essential for the proper functioning of immune cells), etc., play important roles in autoimmune‐related issues in patients receiving hemophilia inhibitors (Hemophilia inhibitory patients) [18, 91].

In conclusion, inhibitory antibodies in patients with severe hemophilia may be associated with or without autoimmune risk‐related genetic factors. However, studies have shown that in most cases, polymorphisms associated with autoimmune diseases produce antibodies or lead to a severe response to treatment factors in these patients. Studying the role of factors and risk factors associated with autoimmune diseases in patients whose treatment is inhibited can effectively improve care and reduce treatment costs for these people.

## Author Contributions

**Fatemeh Zeylabi:** writing – original draft, data curation, investigation, methodology. **Mojtaba Aghaei:** conceptualization, investigation, validation, writing – review and editing, writing – original draft. **Najmaldin Saki:** supervision, conceptualization, writing – review and editing, project administration.

## Ethics Statement

All procedures performed in studies involving human participants were in accordance with the ethical standards of the institutional and/or national research committee and with the 1964 Helsinki declaration and its later amendments or comparable ethical standards.

## Conflicts of Interest

The authors declare no conflicts of interest.

## Transparency Statement

The lead author Najmaldin Saki affirms that this manuscript is an honest, accurate, and transparent account of the study being reported; that no important aspects of the study have been omitted; and that any discrepancies from the study as planned (and, if relevant, registered) have been explained.

## Data Availability

Data sharing not applicable to this article as no datasets were generated or analysed during the current study.

## References

[1] A. Dorgalaleh , G. Dadashizadeh , and T. Bamedi , “Hemophilia in Iran,” Hematology 21, no. 5 (2016): 300–310. 26914731 10.1080/10245332.2015.1125080

[2] C. Santoro , G. Quintavalle , G. Castaman , E. Baldacci , A. Ferretti , and F. Riccardi , ed., Inhibitors in Hemophilia B. Seminars in Thrombosis and Hemostasis. Thieme Medical Publishers, 2018). 10.1055/s-0038-1660817 29925096

[3] E. D. Gomperts , J. Schwarz , S. M. Donfield , et al., “The Importance of Genetic Factors for the Development of Arthropathy: A Longitudinal Study of Children and Adolescents With Haemophilia A,” Thrombosis and Haemostasis 117, no. 02 (2017): 277–285. 27929201 10.1160/TH16-06-0440 PMC8058627

[4] R. Kruse‐Jarres , “Inhibitors: Our Greatest Challenge. Can We Minimize the Incidence?,” Haemophilia 19 (2013): 2–7. 10.1111/hae.12049 23278993

[5] S. Haya , “Prophylactic Treatment in Hemophilic Patients With Inhibitors,” Blood coagulation & Fibrinolysis: An International Journal in Haemostasis and Thrombosis 30, no. Haemophilia and Other Congenital Coagulopathies (2019): 14. 10.1097/MBC.0000000000000823 31517711

[6] M. Cormier , P. Batty , J. Tarrant , and D. Lillicrap , “Advances in Knowledge of Inhibitor Formation in Severe Haemophilia A,” British Journal of Haematology 189, no. 1 (2020): 39–53. 32064603 10.1111/bjh.16377

[7] A. Pavlova , D. Delev , S. Lacroix‐Desmazes , et al., “Impact of Polymorphisms of the Major Histocompatibility Complex Class II, Interleukin‐10, Tumor Necrosis Factor‐α and Cytotoxic T‐Lymphocyte Antigen‐4 Genes on Inhibitor Development in Severe Hemophilia A,” Journal of Thrombosis and Haemostasis 7, no. 12 (2009): 2006–2015. 19817985 10.1111/j.1538-7836.2009.03636.x

[8] C. L. Eckhardt , J. Astermark , S. Q. Nagelkerke , et al., “The Fc Gamma Receptor IIa R131H Polymorphism Is Associated With Inhibitor Development in Severe Hemophilia A,” Journal of Thrombosis and Haemostasis 12, no. 8 (2014): 1294–1301. 24916518 10.1111/jth.12631

[9] G. Ulrich‐Merzenich , A. Hausen , H. Zeitler , G. Goldmann , J. Oldenburg , and A. Pavlova , “The Role of Variant Alleles of the Mannose‐Binding Lectin in the Inhibitor Development in Severe Hemophilia A,” Thrombosis Research 179 (2019): 140–146. 31141731 10.1016/j.thromres.2019.05.005

[10] M. Margaglione and M. Intrieri , ed., Genetic Risk Factors and Inhibitor Development in Hemophilia: What Is Known and Searching for the Unknown. Seminars in Thrombosis and Hemostasis. Thieme Medical Publishers, 2018. 10.1055/s-0038-1660816 29940657

[11] A. F. Karim , A. R. Soltis , G. Sukumar , et al., “Hemophilia A Inhibitor Subjects Show Unique PBMC Gene Expression Profiles That Include Up‐Regulated Innate Immune Modulators,” Frontiers in Immunology 11 (2020): 1219. 32595650 10.3389/fimmu.2020.01219 PMC7303277

[12] J. Oldenburg and A. Pavlova , “Genetic Risk Factors for Inhibitors to Factors VIII and IX,” Haemophilia 12 (2006): 15–22. 17123389 10.1111/j.1365-2516.2006.01361.x

[13] K. Ghosh and S. Shetty , “Immune Response to FVIII in Hemophilia A: An Overview of Risk Factors,” Clinical Reviews in Allergy & Immunology 37 (2009): 58–66. 19148784 10.1007/s12016-009-8118-1

[14] L. Luo , Q. Zheng , Z. Chen , et al., “Hemophilia a Patients With Inhibitors: Mechanistic Insights and Novel Therapeutic Implications,” Frontiers in Immunology 13 (2022): 1019275. 36569839 10.3389/fimmu.2022.1019275 PMC9774473

[15] C. Witmer and G. Young , “Factor VIII Inhibitors in Hemophilia A: Rationale and Latest Evidence,” Therapeutic Advances in Hematology 4, no. 1 (2013): 59–72. 23610614 10.1177/2040620712464509 PMC3629762

[16] M. Franchini and P. M. Mannucci , “Modifiers of Clinical Phenotype in Severe Congenital Hemophilia,” Thrombosis Research 156 (2017): 60–64. 28599169 10.1016/j.thromres.2017.05.038

[17] E. Berntorp and A. D. Shapiro , “Modern Haemophilia Care,” Lancet 379, no. 9824 (2012): 1447–1456. 22456059 10.1016/S0140-6736(11)61139-2

[18] M. Aghaei , R. Khademi , M. A. J. Far , S. S. Bahreiny , A. H. Mahdizade , and N. Amirrajab , “Genetic Variants of Dectin‐1 and Their Antifungal Immunity Impact in Hematologic Malignancies: A Comprehensive Systematic Review,” Current Research in Translational Medicine 72, no. 4 (2024): 103460. 39038414 10.1016/j.retram.2024.103460

[19] G. White , F. Rosendaal , L. Aledort , J. Lusher , C. Rothschild , and J. Ingerslev , “Definitions in Hemophilia: Recommendation of the Scientific Subcommittee on Factor VIII and Factor IX of the Scientific and Standardization Committee of the International Society on Thrombosis and Haemostasis,” Thrombosis and Haemostasis 85, no. 3 (2001): 560. 11307831

[20] D. Chaves , A. Belisário , G. Castro , M. Santoro , and C. Rodrigues , “Analysis of Cytokine Genes Polymorphism as Markers for Inhibitor Development in Haemophilia A,” International Journal of Immunogenetics 37, no. 2 (2010): 79–82. 20082647 10.1111/j.1744-313X.2009.00893.x

[21] J. Astermark , S. M. Donfield , E. D. Gomperts , et al., “The Polygenic Nature of Inhibitors in Hemophilia A: Results From the Hemophilia Inhibitor Genetics Study (HIGS) Combined Cohort,” Blood 121, no. 8 (2013): 1446–1454. 23223434 10.1182/blood-2012-06-434803 PMC3578958

[22] N. Mitsuiki , C. Schwab , and B. Grimbacher , “What Did We Learn From CTLA‐4 Insufficiency on the Human Immune System?,” Immunological Reviews 287, no. 1 (2019): 33–49. 30565239 10.1111/imr.12721

[23] A. I. Mansour , O. G. Behairy , E. R. Abd Almonaem , R. M. Abd‐Rabuh , and I. A. E. Ahmed , “Association of Interleukin (IL)‐4 Variable Number of Tandem Repeats (VNTRs) and IL‐4‐590 Promoter Polymorphisms With Susceptibility to Pediatric Autoimmune Hepatitis Type 1,” Cytokine 110 (2018): 243–247. 29396050 10.1016/j.cyto.2018.01.009

[24] I. Wieland , C. Wermes , B. Eifrig , et al., “Inhibitor‐Immunology‐Study,” Hämostaseologie 31, no. S 01 (2011): S57–S60. 22057736

[25] G.‐R. Kim , S. Lim , J.‐U. Lee , and J.‐M. Choi , “The Cytoplasmic Domain of CTLA‐4 Control Autoimmunity via Inducing Regulatory T Cells,” Journal of Immunology 202, no. 1_Suppl (2019): 193.4.

[26] H. Pergantou , I. Varela , O. Moraloglou , et al., “Impact of HLA Alleles and Cytokine Polymorphisms on Inhibitors Development in Children With Severe Haemophilia A,” Haemophilia 19, no. 5 (2013): 706–710. 23607306 10.1111/hae.12168

[27] E. Omoyinmi , P. Forabosco , R. Hamaoui , et al., Association of the IL‐10 Gene Family Locus on Chromosome 1 With Juvenile Idiopathic Arthritis (JIA). 2012. 10.1371/journal.pone.0047673 PMC3475696 23094074

[28] M. Torabizadeh , M. Aghaei , N. Saki , M. A. Vahid , S. Bitaraf , and B. Bandar , “The Association of Nasal and Blood Eosinophils With Serum IgE Level in Allergic Rhinitis and Asthma: A Case‐Control Study,” Health Science Reports 7, no. 11 (2024): e70191. 39512245 10.1002/hsr2.70191 PMC11541053

[29] L. Mu , X. Yin , Y. Yang , et al., “Functional Characterization of a Mannose‐Binding Lectin (MBL) From Nile Tilapia (*Oreochromis niloticus*) in Non‐Specific Cell Immunity and Apoptosis in Monocytes/Macrophages,” Fish & Shellfish Immunology 87 (2019): 265–274. 30654028 10.1016/j.fsi.2019.01.019

[30] A. Tsutsumi , R. Takahashi , and T. Sumida , “Mannose Binding Lectin: Genetics and Autoimmune Disease,” Autoimmunity Reviews 4, no. 6 (2005): 364–372. 16081027 10.1016/j.autrev.2005.02.004

[31] S. S. Bahreiny , A. Ahangarpour , and M. Aghaei , “Circulating Levels of Advanced Glycation End Products in Females With Polycystic Ovary Syndrome: A Meta‐Analysis,” Reproductive and Developmental Medicine 8, no. 2 (2024): 93–100.

[32] M. Farrokhi , M. Dabirzadeh , N. Dastravan , et al., “Mannose‐Binding Lectin Mediated Complement Pathway in Autoimmune Neurological Disorders,” Iranian Journal of Allergy, Asthma and Immunology 15 (2016): 251–256. 27424141

[33] W. Peng , B. Wang , H. Pan , et al., “Association of the interleukin‐10 1082G/A, 819C/T and 3575T/A Gene Polymorphisms With Systemic Sclerosis: A Meta‐Analysis,” Molecular Biology Reports 39 (2012): 6851–6855. 22307790 10.1007/s11033-012-1510-6

[34] V. Bafunno , R. Santacroce , M. Chetta , et al., “Polymorphisms in Genes Involved in Autoimmune Disease and the Risk of FVIII Inhibitor Development in Italian Patients With Haemophilia A,” Haemophilia 16, no. 3 (2010): 469–473. 20015215 10.1111/j.1365-2516.2009.02150.x

[35] H. Sakalli , W. Matrane , Z. Hamzaoui , and B. Oukkache , “Acquired Hemophilia A: Three Cases and Review of the Literature,” Clinical Laboratory 65, no. 9 (2019): 1745–1750. 10.7754/Clin.Lab.2019.190140 31532083

[36] M. Rydzewska , A. Góralczyk , J. Gościk , et al., “Analysis of Chosen Polymorphisms rs2476601 a/G–PTPN22, rs1990760 C/T–IFIH1, rs179247 A/G–TSHR in Pathogenesis of Autoimmune Thyroid Diseases in Children,” Autoimmunity 51, no. 4 (2018): 183–190. 29973096 10.1080/08916934.2018.1486824

[37] M. M. Gorski , K. Blighe , L. A. Lotta , et al., “Whole‐Exome Sequencing to Identify Genetic Risk Variants Underlying Inhibitor Development in Severe Hemophilia A Patients,” Blood 127, no. 23 (2016): 2924–2933. 27060170 10.1182/blood-2015-12-685735

[38] L. Diaz‐Gallo , P. Gourh , J. Broen , et al., “Analysis of the Influence of PTPN22 Gene Polymorphisms in Systemic Sclerosis,” Annals of the Rheumatic Diseases 70, no. 3 (2011): 454–462. 21131644 10.1136/ard.2010.130138 PMC3170726

[39] S. S. Bahreiny , A. Ahangarpour , K. Hoseinynejad , et al., “Association of KCNJ11 (rs5219) Gene Polymorphism With Susceptibility to Gestational Diabetes Mellitus: A Review and Meta‐Analysis,” Iranian Journal of Obstetrics, Gynecology and Infertility 27, no. 1 (2024): 63–79.

[40] K. Budding , J. Van Setten , E. A. Van de Graaf , et al., “The Autoimmune‐Associated Single Nucleotide Polymorphism Within PTPN22 Correlates With Clinical Outcome After Lung Transplantation,” Frontiers in Immunology 9 (2019): 3105. 30705675 10.3389/fimmu.2018.03105 PMC6344400

[41] M. Aghaei , S. S. Bahreiny , Z. D. Zayeri , N. Davari , M. M. Abolhasani , and N. Saki , “Evaluation of Complete Blood Count Parameters in Patients With Diabetes Mellitus: A Systematic Review,” Health Science Reports 8, no. 2 (2025): e70488. 39995796 10.1002/hsr2.70488 PMC11847716

[42] S. S. Bahreiny , A. Ahangarpour , M. Aghaei , R. Mohammadpour Fard , M. A. Jalali Far , and T. Sakhavarz , “A Closer Look at Galectin‐3: Its Association With Gestational Diabetes Mellitus Revealed by Systematic Review and Meta‐Analysis,” Journal of Diabetes and Metabolic Disorders 23, no. 2 (2024): 1621–1633. 39610475 10.1007/s40200-024-01461-z PMC11599495

[43] O. B. J. Corneth , G. M. P. Verstappen , S. M. J. Paulissen , et al., “Enhanced Bruton's Tyrosine Kinase Activity in Peripheral Blood B Lymphocytes From Patients With Autoimmune Disease,” Arthritis & Rheumatology 69, no. 6 (2017): 1313–1324. 28141917 10.1002/art.40059

[44] S. S. Bahreiny , M.‐N. Bastani , M. Aghaei , M. R. Dabbagh , and A. H. Mahdizade , “Circulating Galectin‐3 Levels in Women With Polycystic Ovary Syndrome: A Meta‐Analysis,” Taiwanese Journal of Obstetrics and Gynecology 63, no. 1 (2024): 37–45. 38216266 10.1016/j.tjog.2023.10.003

[45] J. Mayeux , B. Skaug , W. Luo , et al., “Genetic Interaction Between Lyn, Ets1, and Btk in the Control of Antibody Levels,” Journal of Immunology 195, no. 5 (2015): 1955–1963. 10.4049/jimmunol.1500165 PMC4546901 26209625

[46] L. J. Crofford , L. E. Nyhoff , J. H. Sheehan , and P. L. Kendall , “The Role of Bruton's Tyrosine Kinase in Autoimmunity and Implications for Therapy,” Expert Review of Clinical Immunology 12, no. 7 (2016): 763–773. 26864273 10.1586/1744666X.2016.1152888 PMC5070917

[47] L. A. Honigberg , A. M. Smith , M. Sirisawad , et al., “The Bruton Tyrosine Kinase Inhibitor PCI‐32765 Blocks B‐Cell Activation and Is Efficacious in Models of Autoimmune Disease and B‐Cell Malignancy,” Proceedings of the National Academy of Sciences 107, no. 29 (2010): 13075–13080. 10.1073/pnas.1004594107 PMC2919935 20615965

[48] S. Delignat , J. Russick , B. Gangadharan , et al., “Prevention of the Anti‐Factor VIII Memory B‐Cell Response by Inhibition of Bruton Tyrosine Kinase in Experimental Hemophilia A,” Haematologica 104, no. 5 (2019): 1046–1054. 30545924 10.3324/haematol.2018.200279 PMC6518880

[49] L. L. Jardim , D. G. Chaves , A. C. O. Silveira‐Cassette , et al., “Immune Status of Patients With Haemophilia A Before Exposure to Factor VIII: First Results From the HEMFIL Study,” British Journal of Haematology 178, no. 6 (2017): 971–978. 28836262 10.1111/bjh.14799

[50] L. M. M. de Oliveira , L. L. Jardim , M. A. P. Santana , et al., “Effect of the First Factor VIII Infusions on Immunological Biomarkers in Previously Untreated Patients With Hemophilia A From the HEMFIL Study,” Thrombosis and Haemostasis 121, no. 7 (2021): 891–899. 33423244 10.1055/s-0040-1722353

[51] L. Luo , X. An , Y. Wang , et al., “Chemokine CXCL13 Facilitates Anti‐FVIII Inhibitory Antibody Development in Hemophilia A Patients and Murine Models,” International Immunopharmacology 143, no. Pt 2 (2024): 113472. 39471695 10.1016/j.intimp.2024.113472 PMC12117665

[52] Q. Zheng , K. Lin , N. Zhang , Q. Shi , Y. Wu , and Y. Chen , “Anti‐mCD20 in Combination With α‐mCXCL13 Monoclonal Antibody Inhibits Anti‐FVIII Antibody Development in Hemophilia A Mice,” International Immunopharmacology 139 (2024): 112735. 39067397 10.1016/j.intimp.2024.112735

[53] W. Jing , J. A. Schroeder , S. Kumar , et al., “The T Follicular Helper/T Follicular Helper Regulatory Pathway in FVIII Immune Responses in Mice,” Blood 146, no. 8 (2025): 998–1010. 40493881 10.1182/blood.2025029470 PMC12412428

[54] X. Zhang , “IL‐10 Is Involved in the Suppression of Experimental Autoimmune Encephalomyelitis by CD25+CD4+ Regulatory T Cells,” International Immunology 16, no. 2 (2004): 249–256. 14734610 10.1093/intimm/dxh029

[55] J. Li , X. Liu , L. Li , et al., “Correlation Analysis of Gene Polymorphisms and β‐Lactam Allergy,” Journal of Zhejiang University‐SCIENCE B 16, no. 7 (2015): 632–639. 26160721 10.1631/jzus.B1400309 PMC4506954

[56] S. Kodama , M. Davis , and D. L. Faustman , “The Therapeutic Potential of Tumor Necrosis Factor for Autoimmune Disease: A Mechanistically Based Hypothesis,” Cellular and Molecular Life Sciences 62, no. 16 (2005): 1850–1862. 15968469 10.1007/s00018-005-5022-6 PMC11138375

[57] A. Pavlova , D. Delev , S. Lacroix‐desmazes , et al., “Impact of Polymorphisms of the Major Histocompatibility Complex Class II, Interleukin‐10, Tumor Necrosis Factor‐α and Cytotoxic T‐Lymphocyte Antigen‐4 Genes on Inhibitor Development in Severe Hemophilia A,” Journal of Thrombosis and Haemostasis 7, no. 12 (2009): 2006–2015. 19817985 10.1111/j.1538-7836.2009.03636.x

[58] L. G. Ng , A. P. R. Sutherland , R. Newton , et al., “B Cell‐Activating Factor Belonging to the TNF Family (BAFF)‐R Is the Principal BAFF Receptor Facilitating BAFF Costimulation of Circulating T and B Cells,” Journal of Immunology 173, no. 2 (2004): 807–817. 10.4049/jimmunol.173.2.807 15240667

[59] B. S. Doshi , J. Rana , G. Castaman , et al., “B Cell‐Activating Factor Modulates the Factor VIII Immune Response in Hemophilia A,” Journal of Clinical Investigation 131, no. 8 (2021): e142906, 10.1172/JCI142906. 33651716 PMC8262462

[60] A. Schimpl , I. Berberich , B. Kneitz , et al., “IL‐2 and Autoimmune Disease,” Cytokine & Growth Factor Reviews 13, no. 4–5 (2002): 369–378. 12220550 10.1016/s1359-6101(02)00022-9

[61] N. C. Ward , A. Yu , A. Moro , et al., “IL‐2/CD25: A Long‐Acting Fusion Protein That Promotes Immune Tolerance by Selectively Targeting the IL‐2 Receptor on Regulatory T Cells,” Journal of Immunology 201, no. 9 (2018): 2579–2592. 10.4049/jimmunol.1800907 PMC6200646 30282751

[62] H. Haybar , M. Shokuhian , M. Bagheri , N. Davari , and N. Saki , “Involvement of Circulating Inflammatory Factors in Prognosis and Risk of Cardiovascular Disease,” Journal of Molecular and Cellular Cardiology 132 (2019): 110–119. 31102585 10.1016/j.yjmcc.2019.05.010

[63] J. W. Lowenthal , R. H. Zubler , M. Nabholz , and H. R. MacDonald , “Similarities Between Interleukin‐2 Receptor Number and Affinity on Activated B and T Lymphocytes,” Nature 315, no. 6021 (1985): 669–672. 3925347 10.1038/315669a0

[64] W. Liao , J.‐X. Lin , and W. J. Leonard , “IL‐2 Family Cytokines: New Insights Into the Complex Roles of IL‐2 as a Broad Regulator of T Helper Cell Differentiation,” Current Opinion in Immunology 23, no. 5 (2011): 598–604. 21889323 10.1016/j.coi.2011.08.003 PMC3405730

[65] J. D. Miller , S. E. Clabaugh , D. R. Smith , R. B. Stevens , and L. E. Wrenshall , “Interleukin‐2 Is Present in Human Blood Vessels and Released in Biologically Active Form by Heparanase,” Immunology & Cell Biology 90, no. 2 (2012): 159–167. 21606942 10.1038/icb.2011.45 PMC3162067

[66] T. Arkatkar , S. W. Du , H. M. Jacobs , et al., “B Cell‐Derived IL‐6 Initiates Spontaneous Germinal Center Formation During Systemic Autoimmunity,” Journal of Experimental Medicine 214, no. 11 (2017): 3207–3217. 28899868 10.1084/jem.20170580 PMC5679179

[67] N. S. Sherafat , A. Keshavarz , A. Mardi , et al., “Rationale of Using Immune Checkpoint Inhibitors (ICIs) and Anti‐Angiogenic Agents in Cancer Treatment From a Molecular Perspective,” Clinical and Experimental Medicine 25, no. 1 (2025): 238. 40629032 10.1007/s10238-025-01751-7 PMC12238221

[68] S. S. Bahreiny , M.‐N. Bastani , M. R. Dabbagh , et al., “Association Between Ambient Particulate Matter and Semen Quality Parameters: A Systematic Review and Meta‐Analysis,” Middle East Fertility Society Journal 29, no. 1 (2024): 2.

[69] M. J. Smyth , M. Taniguchi , and S. E. A. Street , “The Anti‐Tumor Activity of IL‐12: Mechanisms of Innate Immunity That Are Model and Dose Dependent,” Journal of Immunology 165, no. 5 (2000): 2665–2670. 10.4049/jimmunol.165.5.2665 10946296

[70] B. Everts , R. Tussiwand , L. Dreesen , et al., “Migratory CD103+ Dendritic Cells Suppress Helminth‐Driven Type 2 Immunity Through Constitutive Expression of Il‐12,” Journal of Experimental Medicine 213, no. 1 (2016): 35–51. 26712805 10.1084/jem.20150235 PMC4710198

[71] O. S. Aval , A. Ahmadi , A. J. Hemid Al‐Athari , et al., “Galectin‐9: A Double‐Edged Sword in Acute Myeloid Leukemia,” Annals of Hematology 104, no. 6 (2025): 3077–3090. 40341460 10.1007/s00277-025-06387-x PMC12283773

[72] J. Astermark , J. Oldenburg , A. Pavlova , E. Berntorp , and A. K. Lefvert , “Polymorphisms in the IL10 but Not in the IL1beta and IL4 Genes Are Associated With Inhibitor Development in Patients With Hemophilia A,” Blood 107, no. 8 (2006): 3167–3172. 16380445 10.1182/blood-2005-09-3918

[73] S. S. Bahreiny , A. Ahangarpour , E. Rajaei , M. S. Sharifani , and M. Aghaei , “Meta‐Analytical and Meta‐Regression Evaluation of Subclinical Hyperthyroidism's Effect on Male Reproductive Health: Hormonal and Seminal Perspectives,” Reproductive Sciences 31, no. 10 (2024): 2957–2971. 39168918 10.1007/s43032-024-01676-8

[74] A. H. Mahdizade , S. S. Bahreiny , M.‐N. Bastani , et al., “The Influence of CDKAL1 (rs7754840) Gene Polymorphism on Susceptibility to Gestational Diabetes Mellitus in Pregnant Women: A Systematic Review and Meta‐Analysis,” International Journal of Diabetes in Developing Countries 44, no. 1 (2024): 3–12.

[75] P. J. Hunt , S. E. Marshall , A. P. Weetman , J. I. Bell , J. A. Wass , and K. I. Welsh , “Cytokine Gene Polymorphisms in Autoimmune Thyroid Disease,” Journal of Clinical Endocrinology and Metabolism 85, no. 5 (2000): 1984–1988. 10843185 10.1210/jcem.85.5.6588

[76] C. Ito , M. Watanabe , N. Okuda , C. Watanabe , and Y. Iwatani , “Association Between the Severity of Hashimoto's Disease and the Functional +874A/T Polymorphism in the Interferon‐.GAMMA. Gene,” Endocrine Journal 53, no. 4 (2006): 473–478. 16820703 10.1507/endocrj.k06-015

[77] J. N. Lozier , P. S. Rosenberg , J. J. Goedert , and I. Menashe , “A Case–Control Study Reveals Immunoregulatory Gene Haplotypes That Influence Inhibitor Risk in Severe Haemophilia A,” Haemophilia 17, no. 4 (2011): 641–649. 21362111 10.1111/j.1365-2516.2010.02473.x PMC3120902

[78] F. Hayashi , M. Watanabe , T. Nanba , N. Inoue , T. Akamizu , and Y. Iwatani , “Association of the ‐31C/T Functional Polymorphism in the Interleukin‐1beta Gene With the Intractability of Graves' Disease and the Proportion of T Helper Type 17 Cells,” Clinical and Experimental Immunology 158, no. 3 (2009): 281–286. 19793334 10.1111/j.1365-2249.2009.04034.x PMC2792823

[79] M. S. Fife , E. M. Ogilvie , D. Kelberman , et al., “Novel IL‐6 Haplotypes and Disease Association,” Genes and Immunity 6, no. 4 (2005): 367–370. 15815691 10.1038/sj.gene.6364186

[80] M. N. Plataki , M. I. Zervou , G. Samonis , V. Daraki , G. N. Goulielmos , and D. P. Kofteridis , “Association of the Interleukin‐6 rs1800795 Polymorphism With Type 2 Diabetes Mellitus in the Population of the Island of Crete, Greece,” Genetic Testing and Molecular Biomarkers 22, no. 7 (2018): 448–452. 29957071 10.1089/gtmb.2017.0220

[81] H. Hassab , M. Hanafi , A. Abdelmaksoud , and S. ElSayed , “Impact of Interleukin‐6 and Interleukin‐10 Gene Polymorphisms on Inhibitor Development in Patients With Moderate and Severe Hemophilia a,” Blood 132, no. Suppl 1 (2018): 5026 ‐.

[82] C.‐L. Liu , P. Ye , B. C. Yen , and C. H. Miao , “In Vivo Expansion of Regulatory T Cells With IL‐2/IL‐2 mAb Complexes Prevents Anti‐Factor VIII Immune Responses in Hemophilia A Mice Treated With Factor VIII Plasmid‐Mediated Gene Therapy,” Molecular Therapy 19, no. 8 (2011): 1511–1520. 21468007 10.1038/mt.2011.61 PMC3149174

[83] M. Fichna , M. Żurawek , E. Bratland , et al., “Interleukin‐2 and Subunit Alpha of Its Soluble Receptor in Autoimmune Addison's Disease–An Association Study and Expression Analysis,” Autoimmunity 48, no. 2 (2015): 100–107. 25347332 10.3109/08916934.2014.976628

[84] N. Naderi , F. Ebrahimzadeh , M. Jazebi , A. Namvar , M. Hashemi , and A. Bolhassani , “Polymorphisms in the TGF‐β1 (rs1982037) and IL‐2 (rs2069762, rs4833248) Genes Are Not Associated With Inhibitor Development in Iranian Patients With Hemophilia A,” Hematology 23, no. 10 (2018): 839–843. 29993342 10.1080/10245332.2018.1498168

[85] B. Srivastava , G. F. Mells , H. J. Cordell , et al., “Fine Mapping and Replication of Genetic Risk Loci in Primary Sclerosing Cholangitis,” Scandinavian Journal of Gastroenterology 47, no. 7 (2012): 820–826. 22554193 10.3109/00365521.2012.682090

[86] N. Inoue , M. Watanabe , M. Morita , et al., “Association of Functional Polymorphisms in Promoter Regions of IL5, IL6 and IL13 Genes With Development and Prognosis of Autoimmune Thyroid Diseases,” Clinical and Experimental Immunology 163, no. 3 (2011): 318–323. 21235536 10.1111/j.1365-2249.2010.04306.x PMC3048614

[87] W. Zhu , N. Liu , Y. Zhao , H. Jia , B. Cui , and G. Ning , “Association Analysis of Polymorphisms in IL‐3, IL‐4, IL‐5, IL‐9, and IL‐13 With Graves’ Disease,” Journal of Endocrinological Investigation 33, no. 10 (2010): 751–755. 20332709 10.1007/BF03346682

[88] C. Dong , T. Fu , J. Ji , Z. Li , and Z. Gu , “The Role of Interleukin‐4 in Rheumatic Diseases,” Clinical and Experimental Pharmacology and Physiology 45, no. 8 (2018): 747–754. 29655253 10.1111/1440-1681.12946

[89] T. B. De Souza , T. L. De Souza , C. dos Santos Ferreira , et al., “A Differentially Methylated CpG Site in the IL4 Gene Associated With Anti‐FVIII Inhibitor Antibody Development in Hemophilia A,” BioRxiv (2019): 550566, 10.1101/550566.

[90] Y. M. Huang , L. J. Shih , T. W. Hsieh , et al., “Type 2 Hypersensitivity Disorders, Including Systemic Lupus Erythematosus, Sjögren's Syndrome, Graves’ Disease, Myasthenia Gravis, Immune Thrombocytopenia, Autoimmune Hemolytic Anemia, Dermatomyositis, and Graft‐Versus‐Host Disease, Are THαβ‐Dominant Autoimmune Diseases,” Virulence 15, no. 1 (2024): 2404225. 39267271 10.1080/21505594.2024.2404225 PMC11409508

[91] P. P. G. Mulder , M. Vlig , E. Fasse , et al., “Burn‐Injured Skin Is Marked by a Prolonged Local Acute Inflammatory Response of Innate Immune Cells and Pro‐Inflammatory Cytokines,” Frontiers in Immunology 13 (2022): 1034420. 36451819 10.3389/fimmu.2022.1034420 PMC9703075

